# DFMO Improves Survival and Increases Immune Cell Infiltration in Association with MYC Downregulation in the Pancreatic Tumor Microenvironment

**DOI:** 10.3390/ijms222413175

**Published:** 2021-12-07

**Authors:** Sai Preethi Nakkina, Sarah B. Gitto, Jordan M. Beardsley, Veethika Pandey, Michael W. Rohr, Jignesh G. Parikh, Otto Phanstiel, Deborah A. Altomare

**Affiliations:** 1 Burnett School of Biomedical Sciences, College of Medicine, University of Central Florida, 6900 Lake Nona Blvd., Orlando, FL 32827, USA; Preethi.Nakkina@Knights.ucf.edu (S.P.N.); Jordan.Beardsley@ucf.edu (J.M.B.); MRohr1@knights.ucf.edu (M.W.R.); 2 Ovarian Cancer Research Center, Division of Gynecology Oncology, Department of Obstetrics and Gynecology, Perelman School of Medicine, University of Pennsylvania, Philadelphia, PA 19104, USA; sarah.gitto@pennmedicine.upenn.edu (S.B.G.); Veethika.Pandey@pennmedicine.upenn.edu (V.P.); 3 Center for Cellular Immunotherapies, University of Pennsylvania, Philadelphia, PA 19104, USA; 4 Abramson Cancer Center, Department of Pathology and Laboratory Medicine, Perelman School of Medicine, University of Pennsylvania, Philadelphia, PA 19104, USA; 5 Department of Pathology, Orlando VA Medical Center, 13800 Veterans Way, Orlando, FL 32827, USA; drjigneshgp@yahoo.com; 6 Department of Medical Education, College of Medicine, University of Central Florida, 12722 Research Parkway, Orlando, FL 32826, USA; otto.phanstiel@ucf.edu

**Keywords:** pancreatic ductal adenocarcinoma, polyamine metabolism, tumor microenvironment, immune suppression, MYC, KRAS, c-RAF, DFMO, GW5074

## Abstract

Pancreatic ductal adenocarcinoma (PDAC) has an extremely poor five-year survival rate of less than 10%. Immune suppression along with chemoresistance are obstacles for PDAC therapeutic treatment. Innate immune cells, such as tumor-associated macrophages, are recruited to the inflammatory environment of PDAC and adversely suppress cytotoxic T lymphocytes. KRAS and MYC are important oncogenes associated with immune suppression and pose a challenge to successful therapies. Here, we targeted KRAS, through inhibition of downstream c-RAF with GW5074, and MYC expression via difluoromethylornithine (DFMO). DFMO alone and with GW5074 reduced in vitro PDAC cell viability. Both DFMO and GW5074 showed efficacy in reducing in vivo PDAC growth in an immunocompromised model. Results in immunocompetent syngeneic tumor-bearing mice showed that DFMO and combination treatment markedly decreased tumor size, but only DFMO increased survival in mice. To further investigate, immunohistochemical staining showed DFMO diminished MYC expression and increased tumor infiltration of macrophages, CD86^+^ cells, CD4^+^ and CD8^+^ T lymphocytes. GW5074 was not as effective in modulating the tumor infiltration of total CD3^+^ lymphocytes or tumor progression and maintained MYC expression. Collectively, this study highlights that in contrast to GW5074, the inhibition of MYC through DFMO may be an effective treatment modality to modulate PDAC immunosuppression.

## 1. Introduction

Pancreatic cancers have a dismal five-year survival rate of less than 10% [1]. It is projected to be the second leading cause of cancer-related deaths in the US by the year 2030 [2]. Advances to increase patient survival have been difficult, in part due to the complexity of the pancreatic tumor environment among other factors [2]. Patients frequently present with advanced stage of the disease when curative surgery is typically not an option [2]. Existing therapies result in chemoresistance and newer therapies, such as FOLFIRINOX and gemcitabine/nab-paclitaxel, are associated with adverse side effects and have failed to increase the survival of pancreatic cancer patients by more than 2–4 months [3]. Therefore, there is a need to identify novel therapeutics to tackle this lethal disease.

One major obstacle that contributes to the failure of therapeutics in PDAC has been attributed to the dense desmoplasia in the tumor microenvironment and upregulation of compensatory molecular pathways [4]. In PDAC, the inflammatory environment primarily recruits innate immune cells, such as immature myeloid cells and tumor-associated macrophages, which can modulate the tumor environment and lead to immune suppression [5]. These immune suppressive cells are known to target T cells through a multitude of approaches and prevent T cell infiltration and activation [6]. Targeting polyamine metabolism is a strategy that could potentially modulate the immune suppression in the tumor environment [7].

Previous studies from our laboratory have shown that inhibiting polyamine synthesis and transport in pancreatic tumors using a polyamine biosynthesis inhibitor (such as DFMO) in combination with Trimer44NMe, a polyamine-based polyamine transport inhibitor (PTI), improves survival of tumor-bearing mice [8]. More recently, the c-RAF inhibitor GW5074 was identified from a high throughput drug screen as a non-polyamine-based polyamine transport inhibitor [9]. This study also showed that GW5074 may have promise in treating pancreatic cancer. The advantage of a non-polyamine-based polyamine transport inhibitor such as GW5074 is that it has the potential to block polyamine import in the presence of high polyamine concentrations, which may increase therapeutic efficacy.

Pertinent to this study, we elected to investigate GW5074 in combination with DFMO. One method commonly employed in PDAC treatment is targeting the RAF effector pathway downstream of KRAS signaling [10]. KRAS signaling modulates a plethora of hallmark tumor pathways, including PDAC tumor initiation, maintenance, desmoplasia, metastasis, immunity, and drug sensitivity [11,12]. c-MYC acts as a cooperative downstream oncogenic factor to KRAS and has been shown to be overexpressed in both primary and metastatic pancreatic tumors [13,14]. c-MYC downregulation has been linked to increased anti-tumor immune response [15]. Moreover, MYC signaling also regulates the activity of ornithine decarboxylase (ODC), which is involved in the rate limiting step of polyamine biosynthesis [16,17]. The inhibitor of ODC, DFMO, has been shown to impair MYC-associated tumor malignancy in various cancers [18,19,20].

We hypothesized that a combinatorial therapeutic approach using DFMO and GW5074 would be more effective at inhibiting pancreatic tumor cell growth and may ultimately impact immune suppression in the tumor microenvironment. While in vitro and initial in vivo studies using the immunocompromised orthotopic xenograft model partially supported the hypothesis, we found that in an immunocompetent model the combination treatment had no effect on survival when compared to DFMO alone. To better understand this inconsistency, we analyzed treated tumors by immunohistochemistry. It was determined that DFMO-treated tumors exhibited an increase in F4/80 (macrophage marker), CD86 (a T cell-costimulatory marker) and infiltration of T lymphocytes into the tumor environment as demonstrated by increased CD3, CD4 and CD8 expression in DFMO-treated tumor groups. DFMO decreased MYC expression, as expected. In contrast, treatment with the c-RAF inhibitor GW5074 alone did not significantly block in vivo PDAC growth and/or progression in the immunocompetent model. Even at higher doses of GW5074, MYC expression was retained and there was no significant change in immune cell infiltration into the PDAC tumors. Collectively, the present study highlights the importance of DFMO treatment in suppressing MYC expression to improve treatment of chemo-resistant PDAC and the overall effectiveness of DFMO as a modulator of the PDAC tumor and immune microenvironment.

## 2. Results

### 2.1. DFMO and DFMO + GW5074 Treatments Decrease Pancreatic Cancer Cell Viability In Vitro

We used KRAS-driven murine PAN 02 and human L3.6pl pancreatic cancer cells to test the effectiveness of DFMO and GW5074 treatments at previously optimized concentrations [9,21,22], alone and in combination, to inhibit pancreatic tumor cell growth. The objective was to test if the combination therapy DFMO + GW5074 may be an alternative strategy for the dual blockade of regulators of ODC transcription, such as MYC and the c-RAF-MEK-ERK signaling cascade.

**Figure 1:**
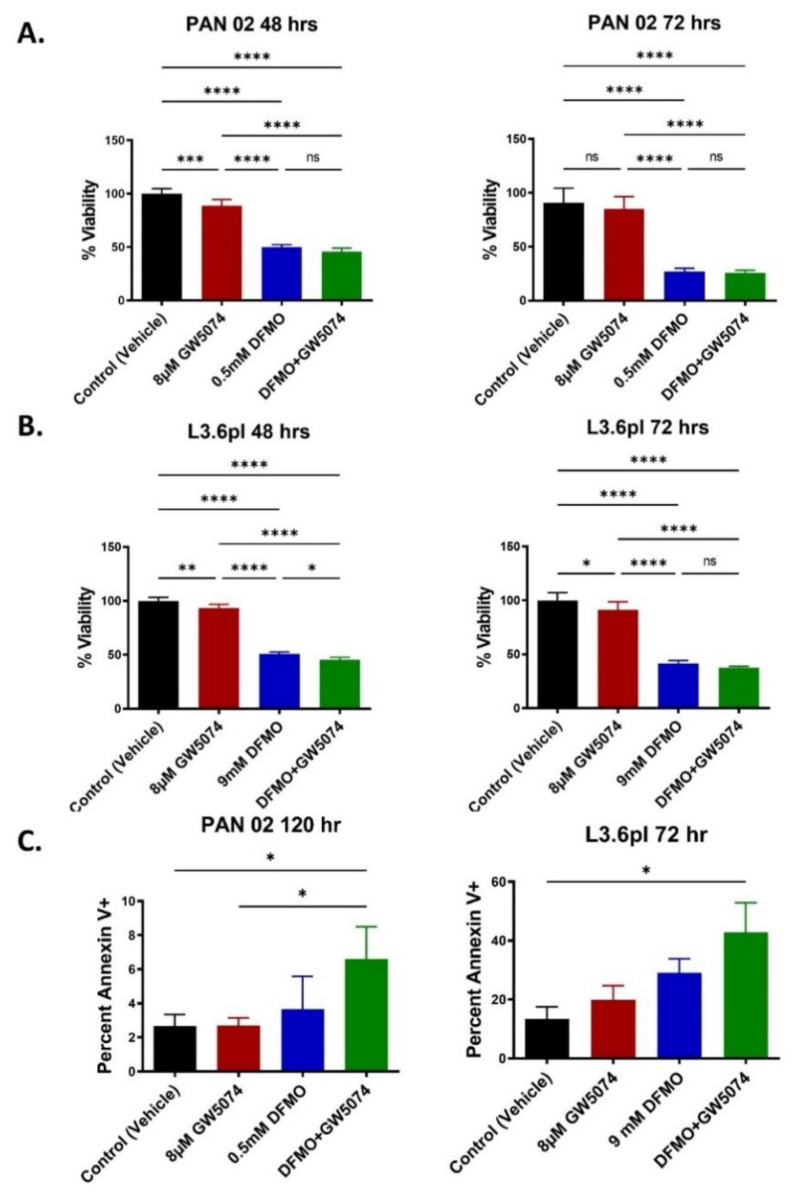
DFMO and DFMO + GW5074 inhibit pancreatic cancer cell viability in vitro. (**A**) The relative growth of PAN 02 cells treated with control, GW5074, DFMO and DFMO + GW5074 for 48 and 72 h were assessed by the MTS assay. (**B**) The relative growth of L3.6pl treated with control, GW5074, DFMO and DFMO + GW5074 for 48 and 72 h were assessed by the MTS assay. (**C**) Flow cytometry analysis of Annexin V+ PAN 02 cells treated for 120 h and L3.6pl cells treated for 72 h revealed a significant increase in apoptotic cells in the DFMO + GW5074 treatment with respect to the control treatment. A one-way ANOVA followed by post hoc Tukey’s test for multiple comparison was used to analyze statistical significance between treatment groups. (*p* < 0.05 [*], *p* < 0.01 [**], *p* < 0.001 [***], *p* < 0.0001 [****]).

PAN 02 cells were treated with control, 0.5 mM DFMO, 8μM GW5074, or a combination of DFMO + GW5074 for 48 h and 72 h. Compared to control and GW5074, there was a significant decrease in PAN 02 cell viability of DFMO (*p* < 0.0001) and DFMO + GW5074 (*p* < 0.0001) treatments at 48 h and 72 h (Figure 1A). In 48 h-treated PAN 02 cells, GW5074 treatment decreased cell viability compared to the control group (*p =* 0.0009). The PAN 02 cells treated with DFMO and DFMO + GW5074 have nearly a 2-fold decrease in viability at 48 h and an approximately 3-fold reduction in cell viability at 72 h when compared to the control group.

L3.6pl cells were treated with control, 9 mM DFMO, 8 μM GW5074 or a combination DFMO + GW5074 for 48 h and 72 h. GW5074 treatment resulted in a modest decrease in cell viability compared to control at the 48 h (*p =* 0.0035) and 72 h (*p =* 0.0492) treatment timepoints. In comparison to control and GW5074-treated L3.6pl cells at 48 h and 72 h treatments, there was a significant decrease in cell viability with DFMO (*p* < 0.0001) and DFMO + GW5074 (*p* < 0.0001) treatments (Figure 1B). The L3.6pl cells treated with DFMO and DFMO + GW5074 have nearly 2-fold decrease in viability at 48 h, and around a 2.5-fold reduction in cell viability at 72 h when compared to the control group.

PAN 02 cells treated for 120 h with DFMO + GW5074 treatment resulted in the most prominent (2.5-fold) and significant (*p* = 0.0366) increase in the percentage of Annexin V+ cells when compared to the control treatment group. The single agents GW5074 (*p* > 0.9999) and DFMO (*p* = 0.8213) did not show any significant changes in comparison to the control group (Figure 1C). L3.6pl cells treated for 72 h with DFMO + GW5074 had a 2-fold increase in apoptotic Annexin V+ cells. Overall, in 72 h-treated L3.6pl cells, DFMO + GW5074 treatment resulted in the most dramatic (2-fold) and significant (*p* = 0.0032) increase in the percentage of Annexin V+ cells when compared to the control treatment group. In contrast, the single agents GW5074 (*p* = 0.7572) and DFMO (*p* = 0.2084) did not show any significant changes in comparison to the control group (Figure 1C).

These findings support the hypothesis that GW5074 could work with DFMO to increase pancreatic cancer cell apoptosis.

### 2.2. DFMO Alone Markedly Increases Survival of Pancreatic Tumor-Bearing Mice in Contrast with GW5074 across Multiple Dosing and Tumor Seeding Strategies

To test the DFMO + GW5074 therapeutic efficacy in vivo, we injected human L3.6pl tumor cells into the pancreas of athymic nude mice. One week post orthotopic tumor cell implantation, mice were treated for two weeks with control, GW5074 (1 mg/kg), DFMO (1% *w*/*v*) and DFMO + GW5074. The doses of GW5074 and DFMO tested were based on published studies and preliminary tolerability studies we conducted [8,10].

**Figure 2:**
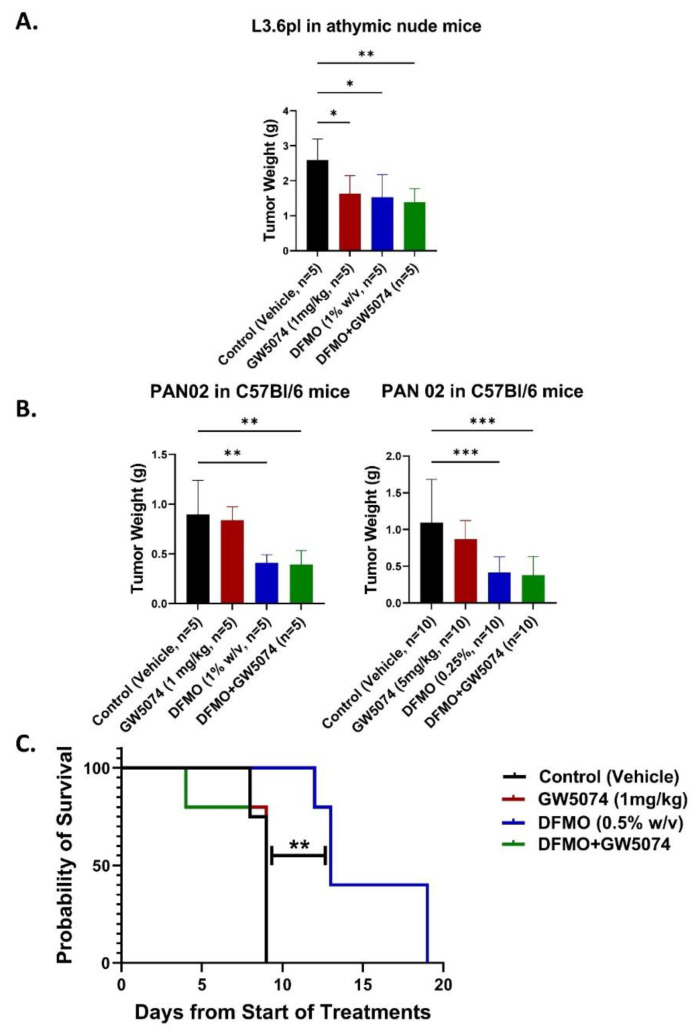
Decreased tumor weights with various in vivo dosing strategies, but DFMO alone markedly increases survival of pancreatic tumor-bearing mice in contrast with GW5074 treatment. (**A**) Fixed-term study showing treatment-specific tumor weights of L3.6pl tumor-bearing athymic nude mice. One week post orthotopic tumor cell implantation (*n* = 5 mice per treatment group), mice were treated for two weeks with control, GW5074 (1 mg/kg), DFMO (1% *w*/*v*) and DFMO + GW5074. (**B**) Fixed-term study showing treatment-specific tumor weights of PAN 02 tumor-bearing mice. Left, two weeks post orthotopic tumor cell implantation (*n* = 5 mice per treatment group), mice were treated for two weeks with control, GW5074 (1 mg/kg), DFMO (1% *w*/*v*) and DFMO + GW5074. Right, one week post orthotopic tumor cell implantation (*n* = 10 mice per treatment group), mice were treated with control, GW5074 (5 mg/kg), DFMO (0.25% *w*/*v*) and DFMO + GW5074 for four weeks. (**C**) Kaplan–Meier curves depicting survival of PAN 02 tumor bearing mice. Three weeks post orthotopic tumor cell implantation (*n* = 4–5 mice per treatment group), mice were treated with control, GW5074 (1 mg/kg), DFMO (0.5% *w*/*v*) and DFMO + GW5074 for up to two weeks. DFMO (0.5% *w*/*v*)-treated PAN 02 tumor-bearing mice have significantly higher survival compared to control mice as determined by log rank test (*p* = 0.005) and median survival (13 days in 0.5% DFMO treated mice versus 9 days in control treated mice). Significance of survival differences was calculated using a log-rank test. A one-way ANOVA followed by post hoc Dunnett’s test for multiple comparison was used to analyze statistical significance between mean tumor weights compared to vehicle group. (*p* < 0.05 [*], *p* < 0.01 [**], *p* < 0.001 [***]).

A fixed termination (fixed term) study where all mice were collected after two weeks of receiving treatment was performed. DFMO and GW5074 monotherapies resulted in a significant decrease in tumor weights when compared to control (*p* = 0.0185 and *p* = 0.0347, respectively). DFMO + GW5074 combination therapy also resulted in a significant decrease in tumor weights when compared to control-treated group (*p* = 0.0083) (Figure 2A). DFMO and GW5074 individually and in combination decreased tumor weights in the L3.6pl in vivo model, indicating an improved effect of the DFMO + GW5074 combination over single agents.

To test the DFMO + GW5074 therapeutic efficacy in an immunocompetent in vivo model, we injected murine PAN 02 tumor cells into the pancreas of C57Bl/6 immune-competent mice. Two weeks after tumor implantation, the first series of in vivo studies used GW5074 injected intraperitoneally daily, 5 times per week, and DFMO was provided continuously in the drinking water for two weeks. A fixed-term study where all mice were collected after two weeks of receiving GW5074 (1 mg/kg) and DFMO (1% *w*/*v*) was performed. DFMO monotherapy and the DFMO + GW5074 combination resulted in a significant decrease in tumor weights when compared to control (*p* = 0.0083 and *p* = 0.0062, respectively) (Figure 2B). To further improve the effectiveness of GW5074 to enhance the overall combinatorial effect of the DFMO + GW5074 treatment in vivo, we used a higher dose of GW5074 (5 mg/kg) in combination with a lower dose of DFMO (0.25% *w*/*v*) to treat pancreatic tumor-bearing mice. Tumor-bearing mice were treated one week after tumor implantation. Animals were treated with control, GW5074 (5 mg/kg), DFMO (0.25% *w*/*v*) or DFMO + GW5074 for four weeks. We performed a fixed-term study to determine therapeutic efficiency, molecular markers and tumor environment changes. Significantly lower tumor weights in comparison to control-treated mice were noticed in DFMO (*p* = 0.0010) and DFMO + GW5074 (*p* = 0.005)-treated mice (Figure 2B). With the 5 mg/kg GW5074 and 0.25% *w*/*v* DFMO dose fixed-term study, animals treated with the higher concentration of GW5074 succumbed/deteriorated in health at a rapid pace compared to the remaining treatment groups and were euthanized prior to the fixed-term study’s end point.

For survival study, PAN 02 cells were allowed to grow for a period of 3 weeks prior to treatment in C57Bl/6 mice. A survival study showed that among pancreatic tumor-bearing mice treated with control, GW5074 (1 mg/kg), DFMO (0.5% *w*/*v*), and DFMO + GW5074 for two weeks, only single-agent DFMO treatment resulted in a significant increase in survival compared to the control group (*p* = 0.005) (Figure 2C). DFMO treatment resulted in a 1.4-fold increase in the median survival of tumor-bearing mice, when compared to control, GW5074 and DFMO + GW5074-treated groups. Of note, the positive effect of the combined treatment of DFMO and GW5074 that was observed from the in vitro findings (Figure 1) were not replicated in the immunocompetent in vivo animal model.

In vivo studies were conducted in different models, with different tumor seeding time durations and treatment doses to determine if there was any advantage to the single or combination treatments. For the survival study, we implemented a longer 3-week tumor seeding prior to treatment, which resulted in a more aggressively growing tumor model. It was clear that DMFO treatment alone, even under these stringent conditions, was the only treatment that resulted in improved survival in contrast to all other treatment groups that succumbed to pancreatic cancer by day 10 of treatment.

### 2.3. Histology Reveals Cellular Differences in DFMO Treated Pancreatic Tumors

**Figure 3:**
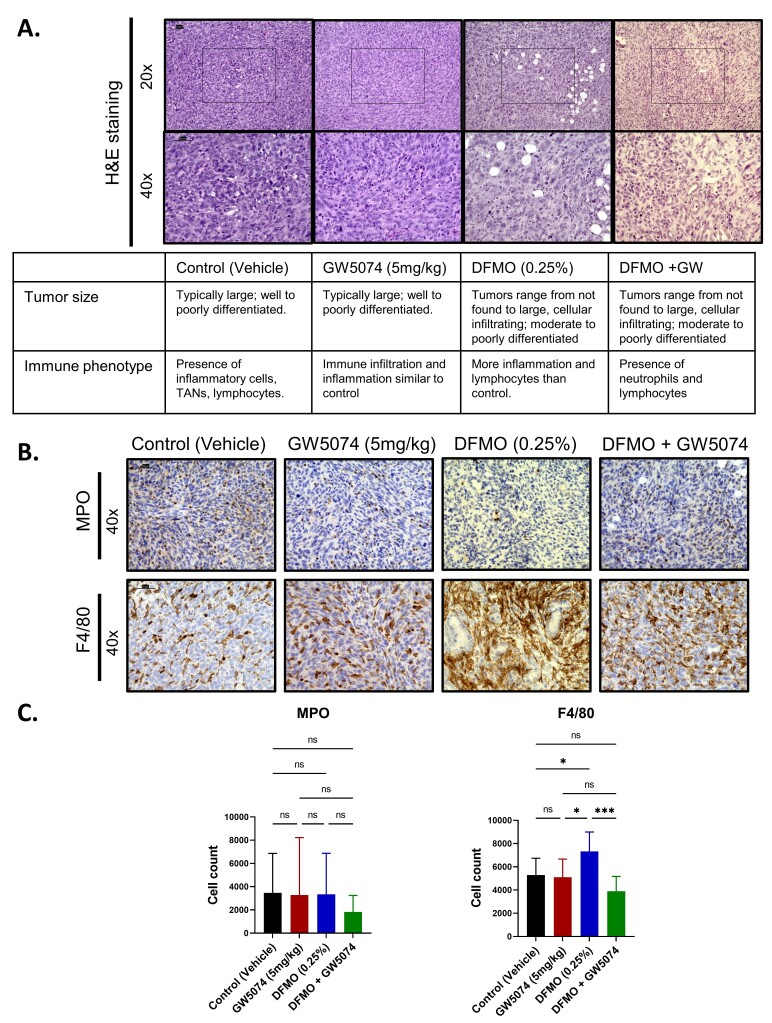
Histological assessment revealed cellular and molecular differences in DFMO-treated pancreatic tumors. (**A**) Tumor sections correspond to mice used for fixed-term studies in Figure 2A, right. These were PAN 02-injected mice treated after one week with control, GW5074 (5 mg/kg), DFMO (0.25% *w*/*v*) and DFMO + GW5074 for a total of four weeks. Hemotoxylin and Eosin (H&E)-stained sections show changes in tumor phenotype and microenvironment, as depicted in the 20× and 40× images (40× corresponds to the boxed regions in the 20× images). Scale bars correspond to 50 μm. Tabulated comments summarize broad cellular changes observed in each treatment group by histological assessment (TAN refers to tumor associated neutrophils). (**B**) Representative immunohistochemistry images of MPO and F4/80-stained DFMO-treated pancreatic tumor sections imaged at 40× magnification. Scale bars correspond to 50 μm. (**C**) Quantification of MPO and F4/80-positive cells across treatment groups. A one-way ANOVA followed by post hoc Tukey’s test for multiple comparison was used to analyze statistical significance between treatment groups (*p* < 0.05 [*], *p* < 0.001 [***]).

To better understand the context for the success of the DFMO treatment strategy in comparison to the other treatments, tumors sections were evaluated for molecular and cellular changes in the tumor microenvironment with respect to treatment groups. Tumor specimens from control, GW5074 (5 mg/kg), DFMO (0.25%), and DFMO + GW5074-treated tumor-bearing mice were first assessed using hematoxylin and eosin (H&E) staining (Figure 3A). Histological assessment by a trained pathologist in a blinded manner revealed tumor regions were consistent with the tumor weights shown in Figure 3A. Additionally, H&E staining revealed the presence of immune infiltrate and inflammatory cells in the tumor microenvironment with potential variations between treatment groups.

Because inflammatory cells commonly present in the tumor environment such as neutrophils and macrophages can either help or hinder anti-tumor immunity in pancreatic cancer, their presence could be linked to therapy-induced inflammation in the tumor microenvironment [21,22]. We then used immunohistochemistry to compare across the treatment groups the differences in the infiltration of neutrophils evaluated by expression of myeloperoxidase (MPO) and macrophages by F4/80 staining (Figure 3B). Quantification revealed neutrophil presence in the tumor but no significant differences in the infiltration of neutrophils based on treatment (*p =* 0.6082). In contrast, DFMO-treated tumors had significantly higher macrophage infiltration when compared to control (*p =* 0.0385), GW5074 (*p =* 0.0126) and DFMO + GW5074 (*p =* 0.0002) treatment groups (Figure 3B,C).

### 2.4. Increased T Cell Infiltration in DFMO Treated Pancreatic Tumors

**Figure 4:**
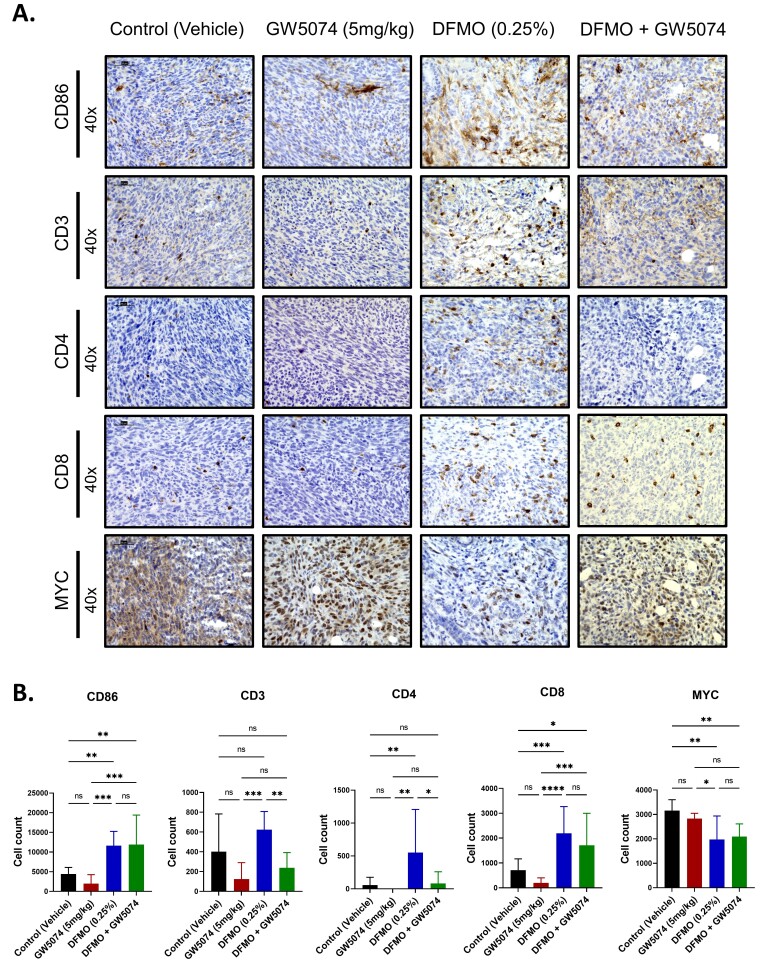
Increased T-cell infiltration in DFMO-treated pancreatic tumors. Tumor sections from PAN 02-injected mice treated after one week with control, GW5074 (5 mg/kg), DFMO (0.25% *w*/*v*) and DFMO + GW5074 for a total of four weeks. (**A**) Representative immunohistochemistry images of CD86, CD3, CD4, CD8 and MYC-stained pancreatic tumor sections imaged at 40× magnification. Scale bars correspond to 50 μm. (**B**) Quantification of CD86, CD3, CD4, CD8 and MYC-positive cells across treatment groups. A one-way ANOVA followed by post hoc Tukey’s test for multiple comparison was used to analyze statistical significance between treatment groups. (*p* < 0.05 [*], *p* < 0.01 [**], *p* < 0.001 [***], *p* < 0.001 [****]).

The next objective was to determine if the inflammation in DFMO-treated pancreatic tumors is associated with T-cell stimulation and infiltration. We assessed the expression of CD86, a T cell co-stimulatory marker present on antigen presenting cells (APCs). CD86 expression was significantly higher in DFMO (*p =* 0.0036) and DFMO + GW5074 (*p =* 0.0043)-treated tumors in comparison to the control-treated group (Figure 4A,B). Both DFMO and DFMO + GW5074-treated tumors showed nearly a 2-fold increase in CD86 expression in comparison to the control-treated group, and an approximately 6-fold increase in CD86 expression in comparison to GW5074-treated tumors.

Increased expression of CD86 could indicate an increased likelihood of T-cell stimulation and subsequently T-cell infiltration. DFMO-treated tumors had significantly higher infiltration of CD3^+^ T lymphocytes when compared to GW5074 (*p =* 0.0007) and DFMO + GW5074 (*p =* 0.01) treatment groups, and a 1.5-fold increase in CD3+ T lymphocytes infiltration when compared to the control-treated group. (Figure 4A,B). Additionally, we assessed the infiltration of CD4^+^ and CD8^+^ T lymphocytes in the tumor microenvironment. DFMO-treated tumors show a significantly higher infiltration of CD4^+^ T cells when compared to control (*p =* 0.0052), GW5074 (*p =* 0.0014) and DFMO + GW5074 (*p =* 0.0153) treatment groups (Figure 4A,B). Compared to control-treated tumors, DFMO (*p =* 0.0007) and DFMO + GW5074 (*p =* 0.0369)-treated groups also showed significantly higher infiltration of CD8^+^ T cells, whereas GW5074 (*p =* 0.4985) showed no significant increase in CD8^+^ T cells (Figure 4A,B).

Overall, cellular changes outlined in Figure 4A,B show an increase in expression of markers associated with anti-tumor activity (CD86, CD3, CD4 and CD8) in DFMO and DFMO + GW5074-treated tumors. Low expression of these markers in control-treated tumors was comparable to the GW5074-treated group, with GW5074-treated tumors showing no significant difference in expression of CD86 (*p =* 0.6104), CD3 (*p =* 0.1202), CD4 (*p =* 0.9727) and CD8 (*p =* 0.4985) in comparison to control-treated tumors. These changes in the tumor microenvironment provided a possible explanation for DFMO’s success in improving the survival of pancreatic tumor-bearing mice and failure of GW5074 and DFMO + GW5074 in vivo.

Another result associated with the increased infiltration of anti-tumor immune cells in the DFMO-treated tumor microenvironment was DFMO-associated MYC downregulation. We assessed MYC expression by immunohistochemistry and found decreased MYC expression in DFMO (*p =* 0.0017) and DFMO + GW5074 (*p =* 0.0062) groups and GW5074-treated tumors maintained MYC expression (*p =* 0.7012) in comparison to control-treated tumors. (Figure 4A,B). In DFMO and DFMO + GW5074 treatment groups, DFMO-associated MYC downregulation was associated with the infiltration of CD8^+^ T lymphocytes in the tumor microenvironment. However, DFMO alone resulted in increased infiltration of total T lymphocytes in the tumor microenvironment assessed by CD3 expression.

## 3. Discussion

In the present study, we used DFMO to target ODC (a MYC transcriptional target) to inhibit MYC expression and therefore MYC-mediated tumorigenesis in PDAC. Earlier studies in genetic mouse models of KRAS mutant pancreatic cancer showed the potential of using DFMO as a chemoprevention agent [20]. Additionally, DFMO has shown preclinical success in various cancer models [16,17,23,24,25,26]. Most notably, DFMO has been successfully used in multiple clinical trials to treat neuroblastoma [16,17,27]. In pancreatic cancer, however, only a handful of preclinical studies have reported using DFMO [8,25]. Mohammed and colleagues were able to decrease the progression of precursor pancreatic neoplastic lesions to PDAC using DFMO as a chemoprevention agent [25]. However, studies evaluating the effectiveness of DFMO as a pancreatic cancer therapeutic are lacking.

A prior study from our group reported that DFMO increased the survival of mice with PDAC tumors when combined with a competitive, polyamine-based polyamine transport inhibitor, Trimer44NMe [8]. The present study tests a novel noncompetitive, non-polyamine-based polyamine transport inhibitor and small molecule inhibitor of RAF, GW5074, which has been shown to be effective in diverse studies, including mediating neuroprotection and alleviating epithelial to mesenchymal transition in a preclinical lung cancer model [28,29]. We tested if GW5074 could be repurposed as a pancreatic cancer therapeutic. GW5074 was identified as a novel non-polyamine-based PTI and inhibits the growth of L3.6pl cell-derived xenografts in immune-deficient Nod Scid Gamma (NSG) mice [9]. To our knowledge this is the only study which has tested GW5074 as a pancreatic cancer therapeutic.

The premise for this study was that we could leverage a combination treatment of DFMO and GW5074 to effectively treat pancreatic cancer. The rationale for using these inhibitors was based on their importance in targeting hallmark signaling pathways in pancreatic cancer and our previous experiences in combination treatments involving DFMO. Indeed, our in vitro results were promising for a potential improvement over that of single agents in diminishing the tumor cell viability of both human L3.6pl pancreatic tumors and mouse PAN 02 pancreatic tumor cells. Additionally, the combination of DFMO and GW5074 showed a significant decrease in tumor weight in vivo in the immunocompromised L3.6pl nude mice model.

However, the efficacy of GW5074 and its action in combination with DFMO could not be replicated in a syngeneic mouse tumor model using orthotopically injected PAN 02 cells (Figure 2A,C). Multiple challenges exist for KRAS targeting via RAF inhibition by GW5074. For example, we encountered solubility issues with GW5074, which originally limited the dosage that could be used with DMSO as a vehicle. When we switched vehicle, we could increase the dose of GW5074, but then we encountered difficulties, with the higher dose having detrimental effects by increasing the tumor growth rate rather than decreasing it.

Additionally, prior studies showed unanticipated signaling pathway effects by targeting this pathway. For example, drug-bound c-RAF can interact with unbound c-RAF, resulting in a paradoxical dimerization event that stimulates kinase activity and downstream signaling [30]. Alternatively, activation of the ERK5 cascade can phosphorylate MYC, compensating for RAF inhibition [31]. Paradoxical dimerization of c-RAF and alternate compensatory signaling cascades, such as possible signaling through other RAF isoforms, could explain the maintenance of MYC expression in GW5074 treated murine PAN 02 tumors and failure to improve survival of pancreatic tumor bearing mice.

Since signaling mechanisms in tumor cells can be complicated to delineate, and because we specifically detected problems with the combination treatment in the immunocompetent mouse model, we decided to test a possible explanation for this paradox by analyzing how the treatments affected immune cell populations and differential immune cell infiltration mediated by individual treatments. In particular, we found that DFMO is coupled with the downregulation of MYC-associated immune suppression in DFMO and DFMO + GW5074 groups, likely increasing the infiltration of CD4^+^ and CD8^+^ T lymphocytes. However, there could be mitigating factors introduced by GW5074 that could further modulate the effects of DFMO in the combination treatment group. In this study, we found that standalone treatment with single-agent DFMO increased the infiltration of total CD3^+^ T lymphocytes into tumors, and even at different test doses demonstrated consistent therapeutic efficacy by improving the survival of tumor-bearing mice (Figure 2B and Figure 4B).

The present study shows the effectiveness of DFMO treatment and provides supporting evidence for the importance of diminishing MYC expression to improve PDAC outcomes. KRAS is a hallmark oncogene in PDAC tumorigenesis [11,32], although the co-operative role of MYC is still being elucidated [33]. It is known that KRAS can initiate tumor progression and formation of early-stage PanIN, whereas the absence of functional MYC prevents transition to PDAC [34,35]. MYC has also been shown to regulate anti-tumor immune responses through various mechanisms, such as secretion of CCL9 and IL-23, and expression of PD-L1 and CD47 [15,36].

Consistent with prior investigations, the present study identified that decreased MYC expression was associated with increased T cell infiltration and potential reduction of immune suppression in murine PDAC tumors. CD4^+^ T lymphocytes can help in anti-tumor immunity by aiding pro-inflammatory antigen-presenting cells and supporting cytotoxic CD8^+^ T lymphocytes [37]. Activated CD8^+^ T lymphocytes are capable of cell-contact-dependent cytotoxicity of malignant cells [38,39,40]. Increased T-cell infiltration plays important roles in mediating anti-tumor immunity. Novel findings from the present study revealed that even with the lowest tested dose of 0.25% DFMO, there was markedly increased infiltration of CD4^+^ and CD8^+^ T lymphocytes into the DFMO and combination-treated tumors, and that this was associated with downregulated MYC expression.

Collectively, these results highlight the ability of DFMO to modulate the tumor microenvironment in PDAC, which is a particularly important finding because overcoming the immune suppressive environment is a critical challenge for ensuring successful PDAC therapy. In contrast, while GW5074 showed effectiveness in vitro and in an immune-deficient PDAC model, it did not demonstrate improved survival and immunomodulatory properties in the immunocompetent PDAC murine model. Thus, other combinatorial reagents warrant further testing in combination with DFMO to promote its immunomodulatory properties.

## 4. Materials and Methods

### 4.1. Reagents 

GW5074 was purchased from Selleckchem (Houston, TX, USA). DFMO was provided by P. Woster (Medical University of South Carolina, Charleston, SC, USA).

### 4.2. Cell Culture 

Murine PAN 02 pancreatic tumor cells were obtained from the Division of Cancer Treatment and Diagnosis (DCTD) Tumor Repository (Frederick, MD, USA). Human L3.6pl pancreatic tumor cells were a generous gift from I. Fidler (MD Anderson Cancer Center, Houston, TX, USA). PAN 02 and L3.6pl cells were grown in DMEM or RPMI-1640 media, respectively, with 1% penicillin/streptomycin and 10% fetal bovine serum and incubated at 37 °C in a 5% CO_2_ incubator.

### 4.3. Cell Viability

Final concentrations of respective compounds were 8 μM GW5074 and 9 mM DFMO with L3.6pl cells (1 × 10^3^ cells per well) or 0.5 mM DFMO with PAN 02 cells (2 × 10^3^ cells per well), as previously described [8,9]. Cells were treated by single agents or combinations for 48 h or 72 h, after which the MTS assay was performed using the CellTiter 96 Aqueous One Solution Cell Proliferation Assay kit (Promega, Madison, WI, USA). Cell viability was determined by measuring formazan formation from the 3-(4,5-dimethylthiazol-2-yl)-5-(3-carboxymethoxyphenyl)-2-(4-sulfenyl)-2H tetrazolium, inner salt. The MTS reagent was added after the treatment period, and the plate was incubated for approximately 2 h, followed by measuring relative absorbance at 490 nm. Absorbance values for the MTS assays were compared to the vehicle-treated control, which was set to 100% viability. Six replicate wells were tested. Results are reported as mean ± SD. To compare each mean with every other mean, a one-way ANOVA followed by post hoc Tukey’s test for multiple comparison was used to analyze statistical significance between treatment groups (*p* < 0.05 [*], *p* < 0.01 [**], *p* < 0.001 [***], *p* < 0.0001 [****]).

### 4.4. Flow Cytometry 

L3.6pl or PAN 02 (2 ×10^5^) cells were grown in 10 cm dishes overnight at 37 °C in a 5% CO_2_ incubator. Final concentrations of respective compounds were 8 μM GW5074 and 9 mM DFMO with L3.6pl cells or 0.5 mM DFMO with PAN 02 cells. Cells were treated by single agents or combinations for 72–120 h, following which cells were collected for apoptosis analysis using FITC Annexin V Apoptosis Detection Kit I (BD Biosciences). Cells were washed with PBS and detached with 0.25% Trypsin, 2.21 mM EDTA without sodium bicarbonate (Corning, Manassas, VA, USA) and collected. Cells were centrifuged at 200× *g* for 5 min and washed with PBS. Cells were stained with Annexin V in staining buffer for 15 min on ice. PBS was added to dilute the Annexin V-stained cells and propidium iodide (1 μL per 250 μL total volume) was added. Stained cells were immediately analyzed with a BD FACS Canto flow cytometer (BD Biosciences). Two to three replicate dishes were tested. Results are reported as mean ± SD. To compare each mean with every other mean, a one-way ANOVA followed by post hoc Tukey’s test for multiple comparisons was used to analyze statistical significance between treatment groups. (*p* < 0.05 [*]).

### 4.5. In Vivo Testing 

In vivo experiments were approved by the University of Central Florida Institutional Animal Care and Use Committee and performed in accordance with the Guide for the Care and Use of Laboratory Animals. Human pancreatic L3.6pl tumor cells (~1 × 10^6^ in PBS) were injected into the pancreas of athymic (nu/nu) mice (Charles River Laboratories, NCI, Fredrick, MD, USA). Murine pancreatic PAN 02 cells (~1 × 10^6^ in PBS) were injected into the pancreas of C57Bl/6 mice (Charles River Laboratories, NCI, Fredrick, MD, USA; Jackson Laboratories, Bar Harbor, ME, USA). Seven days after tumor cell inoculation for L3.6pl and up to 1–2 weeks after tumor inoculation for PAN 02, animals were randomized into groups of mice and treated as indicated with vehicle, GW5074 (1 mg/kg or 5 mg/kg injected intraperitoneally), DFMO (0.5% or 0.25% weight/volume (*w/v*) in the drinking water), or a combination of GW5074 and DFMO for a period of two to four weeks where DFMO was administered continuously (water changed weekly) and GW5074 was administered 5 days per week. For survival studies, PAN 02 cells were inoculated for a period 3 weeks prior to treatment in C57Bl/6 mice.

For studies where GW5074 was dosed at 1 mg/kg, a solution of GW5074 was prepared in a vehicle containing a 1% dimethyl sulfoxide solution in PBS. In studies where GW5074 was dosed at 5 mg/kg, a solution of GW5074 was prepared in a manufacturer-recommended vehicle composed of 30% *v/v* Polyethylene Glycol 400, 5% *v/v* Tween 80 and 65% *v/v* D5W (5% *w*/*v* solution of dextrose monohydrate in molecular grade distilled water). Animals were either necropsied and histological specimens were collected at the end of treatment, or animals were observed after treatment end as a part of a survival study until the mouse was euthanized (due to criteria including loss of >20% body weight or severe lethargic behavior). At necropsy, tumors were collected and weighed (*n* = 4–10 mice per group). Results are reported as mean ± SD. To compare each mean to the control mean, a one-way ANOVA followed by post hoc Dunnett’s test for multiple comparison was used to analyze statistical significance between tumor weights. (*p* < 0.05 [*], *p* < 0.01 [**], *p* < 0.001 [***]). For mice enrolled in the survival study (*n* = 4–5 mice per group), the median survival of tumor-bearing mice in each treatment group was calculated. The significance of survival differences was calculated using a log-rank test. (*p* < 0.01 [**]).

### 4.6. Histological Analysis 

Specimens from necropsied mice were fixed in 10% neutral buffered formalin (Surgipath Leica, Buffalo Grove, IL, USA) and embedded in paraffin. Five-micron sections were sliced using a rotary microtome (Leica). SelecTech hematoxylin and eosin reagents (Surgipath, Buffalo Grove, IL, USA) were used for histological staining. For immunohistochemistry, antigen retrieval was performed with either sodium citrate, pH 6.0, or EDTA, pH 9.0 (Leica, Buffalo Grove, IL, USA). Primary antibodies were against MYC (Abcam, Cambridge, MA, USA), MPO (Thermo Fisher Scientific, Waltham, MA, USA) or F4/80, CD86, CD3, CD4, or CD8 (all from Cell Signaling Technology, Danvers, MA, USA). A Bond-Max Immunostainer and Polymer Refine Detection reagents (Leica, Buffalo Grove, IL, USA) were used for staining. Multiple fields of view of each tumor section were imaged across three representative mice from each treatment group on a Keyence BZ-X800 microscope. Antibody detection was quantified using Keyence BZ-X800 analysis software. Results are reported as mean ± SD. To compare each mean with every other mean, a one-way ANOVA followed by post hoc Tukey’s test for multiple comparison was used to analyze statistical significance between treatment groups. (*p* < 0.05 [*], *p* < 0.01 [**], *p* < 0.001 [***], *p* < 0.0001 [****]).

## 5. Conclusions

Overall, the findings here provide evidence that other agents should be evaluated in combination with DFMO as anticancer therapies to improve its effectiveness as a cancer therapeutic. Among the possible targets that could be tested in future studies are additional immunomodulatory therapeutics. Success here would increase the diversity of molecular tools that could be used to increase pancreatic cancer patient survival.
